# Impact of co-blocking the costimulatory signals on immune-related genes after high-risk rabbit corneal allograft using 2nd-generation DNA sequencing technology

**DOI:** 10.1590/1678-4685-GMB-2018-0150

**Published:** 2019-07-18

**Authors:** Hai-Xia Zhao, Xin-Yu Li, Wen-Ying Guan, Xiao-Tong Han

**Affiliations:** 1 Center of Myopia, The Affiliated Hospital of Inner Mongolia Medical University, Hohhot, China

**Keywords:** Costimulatory signal, corneal allograft, immune rejection, second-generation sequencing technology, antibody

## Abstract

The aim of this study was to evaluate the impact and mechanism of co-blocking of costimulatory signals CD28-B7-CD40-CD40L during immune allograft rejection. Forty-eight recipient rabbits were prepared as a high-risk corneal allograft model. After surgery, the animals were randomly divided into: control group, MR1 group, anti-B7 group, and co-blocking group (n=12, each group). Subconjunctival injection was first performed on the allograft surgery day until post-surgery day five. Four weeks later, or when immune rejection occurred, the cornea was sampled to detect and analyze the gene spectrum. The survival time in the co-blocking group was significantly longer than that in the other three groups (*p* < 0.05). Gene expression analysis revealed that the expression of genes associated with immune rejection, interleukin (IL)-1α, IL-1β, intercellular cell adhesion molecule-1, and IL-2 was down-regulated in the co-blocking group, while IL-10 was up-regulated, but the changes in nuclear factor-κB and interferon-γ were not significant. In conclusion, the co-blocking of costimulatory signals can significantly reduce genes that promote corneal allograft rejection. The inhibition of corneal allograft rejection gene expression was significantly enhanced. These gene expression results can explain the conclusion of previous work at the genetic level.

## Introduction

According to statistics, the blindness of 2–3 million patients is caused by corneal disease (Cornea Group of Ophthalmology Society, Chinese Medical Association, 2014), and this disease has become the second leading cause of blindness (Gong *et al.*, 2015). The only cure for corneal disease is corneal allograft (Zhang *et al.*, 2015). Due to the corneal structure, the graft is relatively immune-tolerant and the rejection rate is low, with success rate higher than that of other organs (Niederkorn, 2013; Yin *et al.*, 2014). However, recent studies have shown that although the incidence of postoperative rejection is low, it can still reach 10–20% (Paunicka *et al.*, 2015; Blanco and Saban, 2015; Choi *et al.*, 2015). Furthermore, high risk patients such as those with neovessels, persistent inflammation, and secondary allograft, the incidence of immune rejection may reach 50% (Tan *et al.*, 2012). Therefore, preventing the occurrence of rejection after high-risk corneal allograft and improving post-keratoplasty graft survival have become key concerns for many ophthalmologists (Dohlman *et al.*, 2015).

In the present study, a high-risk rabbit corneal allograft model was established, in which two antibodies were used to co-block CD28-B7 and CD40-CD40L in the costimulatory pathway (Kirk *et al.*, 1997; Ye *et al.*, 2014). Then gene expression changes in the corneal graft were investigated to evaluate the impact of co-blocking CD28-B7 and CD40-CD40L in the costimulatory pathway on allograft rejection at the gene level. The goal of the present study was to compare graft survival time and to analyze gene expression of different stimulation pathways on high-risk rabbit corneal allograft rejection.

## Material and Methods

### Animals

Seventy-two healthy adult New Zealand white rabbits were provided by the Laboratory Animal Center of Inner Mongolia Medical University. Of the total, 48 rabbits were recipients and 24 were donors. Rabbits weighed within 2-3 kg, without male/female limit. The present study was carried out in strict accordance with the recommendations in the Guide for the Care and Use of Laboratory Animals of the National Institutes of Health. The animal use protocol was reviewed and approved by the Institutional Animal Care and Use Committee (IACUC) of Inner Mongolia Medical University. All rabbits were raised in a laboratory, and the shed met the criterion for experimental rabbit general environmental requirements, such as room temperature within 16-26 °C, day temperature differences of ≤4 °C, relative humidity of 40-70%, minimum air changes of 8 h/time, noise of ≤60 db (A), illumination of 100-200 lx, and an alternation of day and night time of 12/12 hours or 10/14 hours. There was no special requirement on feeding and drinking for the experimental animals.

### Establishment of the high-risk corneal allograft model

The suture method was used to induce neovascularization. First, all animals were injected with 20% urethane solution (3-4 mL/kg) (Sun *et al.*, 2016) into the ear vein for anesthesia. Then, 0.4% oxybuprocaine (Sun *et al.*, 2016) hydrochloride eye drops were used for surface anesthesia in the right eyes of rabbits. Lastly, four sutures were uniformly stitched in the partial central corneal lamina area with 0-5 suture, and the stitch length was approximately 5 mm. Approximately two weeks after the neovessels formed, rabbits with neovessels larger than three quadrants were selected as high-risk recipients for corneal allograft. Before performing penetrating keratoplasty, 1% pilocarpine (Zhao *et al.*, 2013) nitrate eye drops were used to narrow the pupils of the right eyes, three times a day, and 0.2% gentamicin (Sun *et al.*, 2016) was used to rinse the conjunctival sac of right eyes. Then, all animals were injected with 20% urethane solution (3-4 mL/kg) into the ear vein for anesthesia, and the rabbits were allowed to lie on the left side. Then, 0.4% oxybuprocaine hydrochloride eye drops were used for surface anesthesia via the right eye of the rabbits. Lastly, routine aseptic operation was conducted after disinfection. Under an operating microscope, one 7.25-mm trephine (diameter) was used to sample the graft from the corneal surface, and one 7.0-mm trephine (diameter) was used to prepare the cornea for the recipient planting bed. A 10-0 nylon suture was used to apply 16 interrupted sutures. After surgery, 800 U of gentamicin was injected (Sun *et al.*, 2016), and 0.5 mL of 0.5% dexamethasone (Sun *et al.*, 2016) mixture was applied into the subconjunctiva of the right eyes. In addition, 1% atropine eye ointment and tobramycin dexamethasone eye ointment (0.3% tobramycin and 0.1% dexamethasone) were also applied (Sun *et al.*, 2016) to the right eye, which was covered with a sterile eye pad. At the second day after surgery, tobramycin dexamethasone eye drops (0.3% tobramycin and 0.1% dexamethasone) were applied three times a day, 0.5% compound of tropicamide eye drops were applied once a day, and tobramycin dexamethasone eye ointment (0.3% tobramycin and 0.1% dexamethasone) was applied once a day.

### Animal grouping and processing

After surgery, the animals were randomly divided into four groups (n=12). Subconjunctival injection was initially performed on the allograft surgery day, and was continued until post-surgery day five. The control group was injected with saline, the animals in the MR1 group were injected with anti-CD40L monoclonal antibody (Abnova, Taipei, Taiwan) at 100 μg/day, the animals in the anti-B7 group were injected with anti-B7 antibody (Abnova) at 100 μg/day, and the animals in the co-blocking group were injected with MR1 antibody and anti-B7 antibody at 100 μg/day (each).

### Postoperative observation

After surgery, neovessels, graft edema, and turbidity were observed under a slit lamp microscope to assess growth of the planting bed. The allograft rejection index (RI, 0-12 points) was recorded, and these three scores were added to determine the RI on the observation day. The scoring method was as follows: Corneal neovascularization (CNV): no CNV, 0 point; one quadrant of CNV, 1 point; two quadrants of CNV, 2 points; three quadrants of CNV, 3 points; four quadrants of CNV, 4 points. Corneal edema: no corneal edema, 0 point; mild edema, 1 point; mild-to-moderate edema, 2 points; subepithelial micro-vesicle, 3 points; bullous keratopathy, 4 points. Corneal turbidity: transparent corneal, 0 point; mild turbidity, 2 points; corneal aggravated turbidity (anterior chamber structure could be identified with two points): obvious turbidity, and anterior chamber structure is vague, three points; corneal has white turbidity, and anterior chamber cannot be observed, four points. When RI ≥6, cornea rejection occurred, which was considered the graft survival time.

### Sampling and specimen preparation

When rejection occurred at four weeks after the allograft, the rabbits were sacrificed using the air embolism method, and the cornea was rapidly separated and stored at -80°C. After extracting and purifying the RNA (Trizol method), sequencing was performed using a Solexa sequencer (Illumina, San Diego, CA, USA), according to the manufacturer’s instructions, and the data were compared and analyzed. The corneal tissue RNA results of the control and experimental groups are presented in Table 1. The results were 2100 RIN ≥7.0 and 28S/18S ≥0.7 of the four samples, which show that the samples are qualified, and the quality of the RNA was in good condition.

**Table 1:** Corneal tissue RNA extraction results.

Number	Sample group	Concentration (μg/μL)	Quantity before purification (μg)	A260/A280	2100RIN	28S/18S	Result
A	MR1 group	0.1163	11.0	2.13	9.4	1.7	qualified
B	Anti-B7 group	0.8998	85.5	2.06	7.0	1.4	qualified
G	Drug combination group	0.0830	7.9	2.12	9.7	2.0	qualified
C	Control group	0.1288	12.2	2.02	7.1	1.2	qualified

Annotation: qualified: 2100 RIN≥7.0 and 28S/18S≥0.7

### Statistical analysis

SPSS13.0 statistical software (SPSS Inc., Chicago, IL, USA) and GraphPad Prism 5.0 (GraphPad Software Inc., San Diego, CA, USA) were used for data analysis. Data are reported as (mean ± SD). Significance was determined with the Log rank *t*-test for comparing post-allograft graft survival time of the four groups; *p* < 0.05 was considered statistically significant. Clustering analysis, GO functional significance, and pathway enrichment analysis were carried out on the differentially expressed genes.

## Results

### Comparison of graft survival time and survival rate

**Figure 1:**
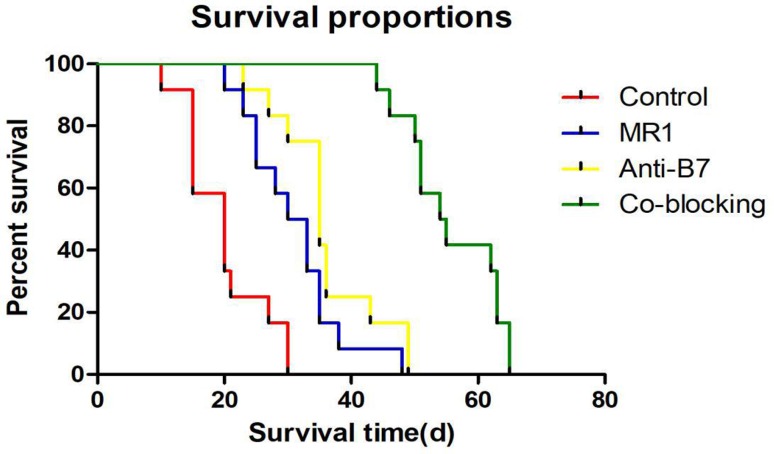
Survival curve of transplanted corneal graft in different groups. (A) survival curve of rabbits in the control group; (B) survival curve of rabbits in the MR1 group; (C) survival curve of rabbits in the Anti-B7 group; (D) survival curve of rabbits in the co-blocking group. Compared with the control group, the co-blocking group displayed a significantly longer allograft survival time. Abscissa: survival time; Ordinate: percent of survival.

The post-allograft graft survival time of the four groups had a normal distribution and the variances were homogeneous. As shown in Figure 1, the survival times of animals in the four groups were the same (*p*<0.0001). Pairwise comparison revealed that grafts in the co-blocking group had a longer mean survival time of 55.75 ± 7.51 days, which was significantly different compared to the other groups (*p*<0.05). Furthermore, the control group exhibited a significantly shorter survival time, when compared to the other groups (19.83 ± 6.68 days, *p*<0.05). The median post-allograft graft survival time of control group, MR1 group, Anti-B7 group, and co-blocking group were 20, 31.5, 35 and 54.5 days, respectively.

### Gene sequencing analysis

The pretreatment results of sequencing data quality are shown in Table 2. The inspection of the base distribution was used to detect the presence of the AT and GC separation phenomenon, which may have been caused by the sequencing or building the library, thereby affecting the subsequent quantitative analysis. In addition, theoretically, G and C bases, as well as A and T bases, on each sequence cycle should be equal and kept stable throughout the whole process. For RNA-seq, there were large fluctuations on the sequencing of few of the bases before each read, due to a random primers amplification bias, which is normal. The results revealed that sample stability and detection rate completely met the requirement.

**Table 2:** Pretreatment results of sequencing data quality.

Sample	Raw reads	Raw bases	Clean bases	Valid ratio (base)	Q30 (%)	GC content (%)
MR1 group	34000000	4250000000	4214703900	99.16%	90.01%	55.00%
Anti-B7group	31869184	3983648000	3950967418	99.17%	90.35%	54.00%
Drug combination group	34000000	4250000000	4216166011	99.20%	90.83%	53.00%
Control group	30478448	3809806000	3777116010	99.14%	89.37%	53.00%

Q30: percentage of bases with Phred values greater than 30 of total base number.

General requirements are of less than 85%.

**Figure 2:**
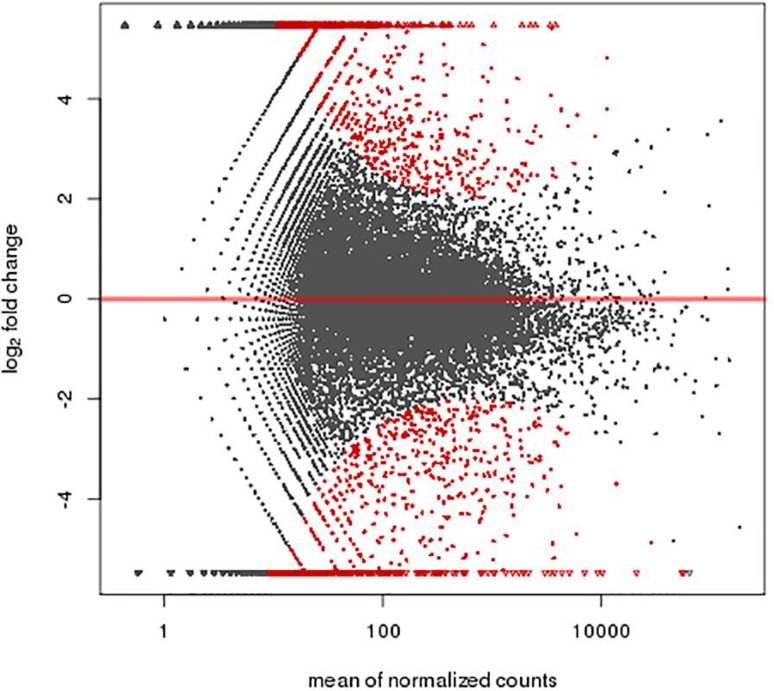
A total of 1,463 genes were differentially expressed between the MR1 group and control group, among which 741 genes were found up-regulated and 722 genes were down-regulated. Abscissa: mean of normalized counts; Ordinate: log_2_ fold change.

A total of 1,463 genes were found differentially expressed between the MR1 group and control group, among which 741 genes were up-regulated and 722 down-regulated. Furthermore, 1,367 genes were differentially expressed between the anti-B7 group and control group, among which 618 genes were up-regulated and 749 down-regulated. A total of 1,325 genes were differentially expressed between the co-blocking group and control group, among which 273 genes were up-regulated and 1,052 down-regulated (Figures 2-4). A total of 1,527 genes were differentially expressed between the co-blocking group and MR1 group, among which 230 genes were up-regulated and 1,297 down-regulated. In addition, 1,739 genes were differentially expressed between the co-blocking group and anti-B7 group, among which 166 genes were up-regulated and 1,573 down-regulated (Figures 5 and 6). The default screening difference was set as *p*≤0.05.

**Figure 3 f3:**
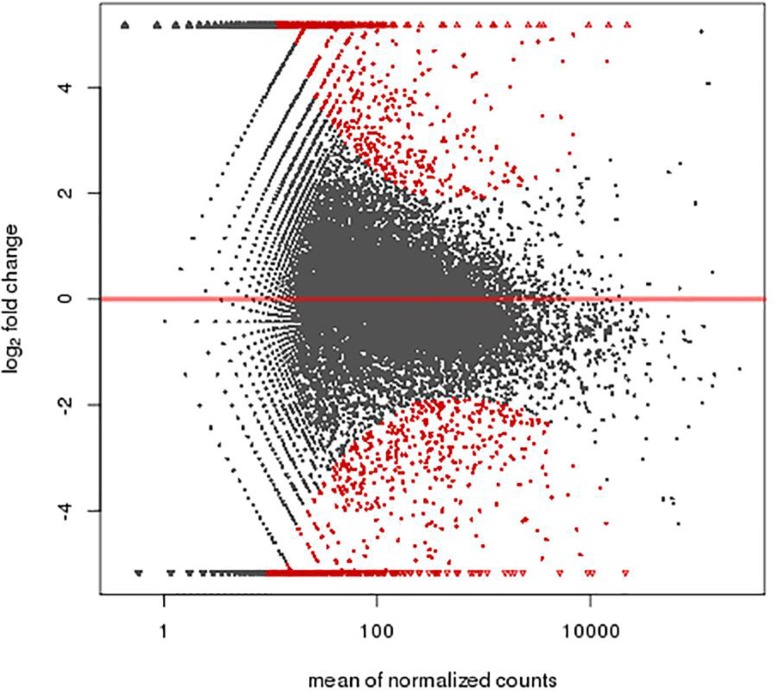
A total of 1,367 genes were differentially expressed between the anti-B7 group and control group, among which 618 genes were up-regulated and 749 down-regulated. Abscissa: mean of normalized counts; Ordinate: log_2_ fold change.

**Figure 4:**
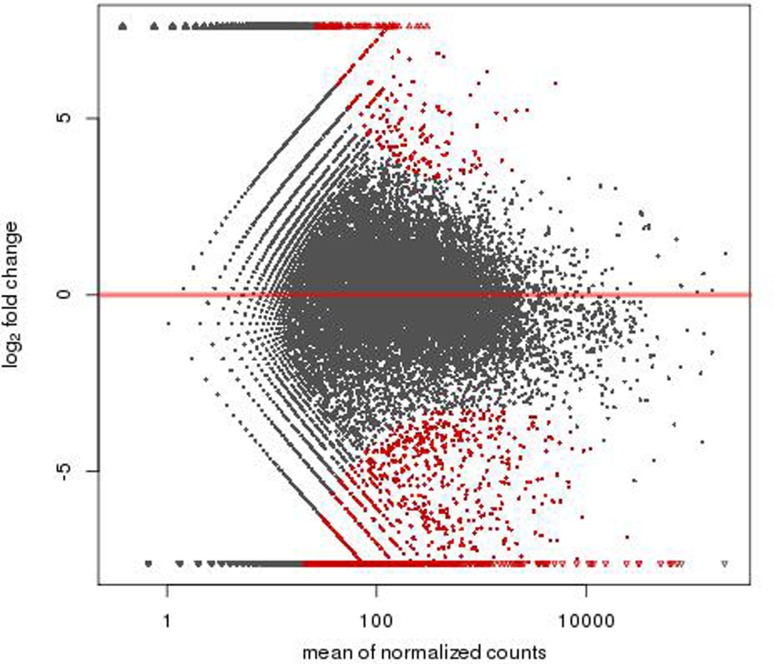
A total of 1,325 genes were differentially expressed between the co-blocking group and control group, among which 273 genes were up-regulated and 1,052 down-regulated. Abscissa: mean of normalized counts; Ordinate: log_2_ fold change.

**Figure 5:**
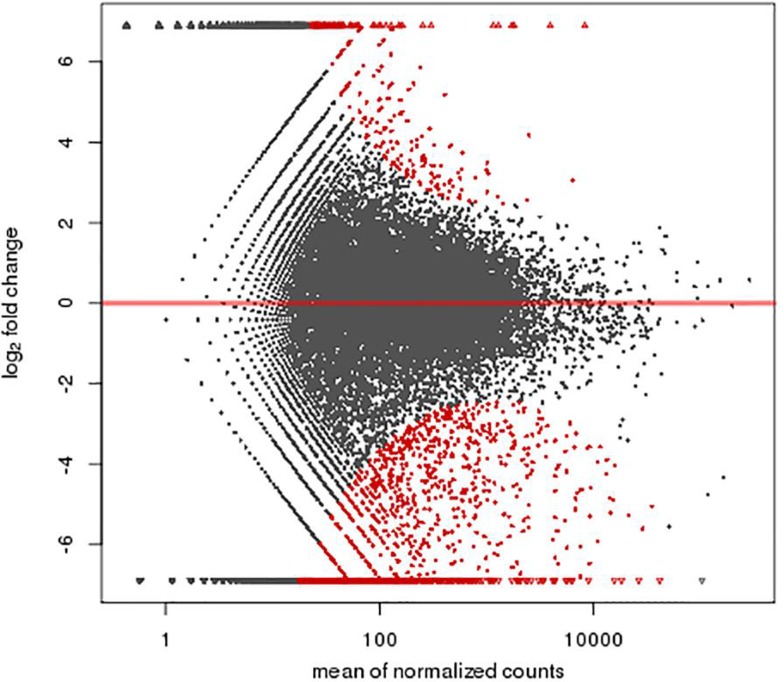
A total of 1,527 genes were differentially expressed between the co-blocking group and MR1 group, among which 230 genes were up-regulated and 1,297 down-regulated. Abscissa: mean of normalized counts; Ordinate: log_2_ fold change.

**Figure 6:**
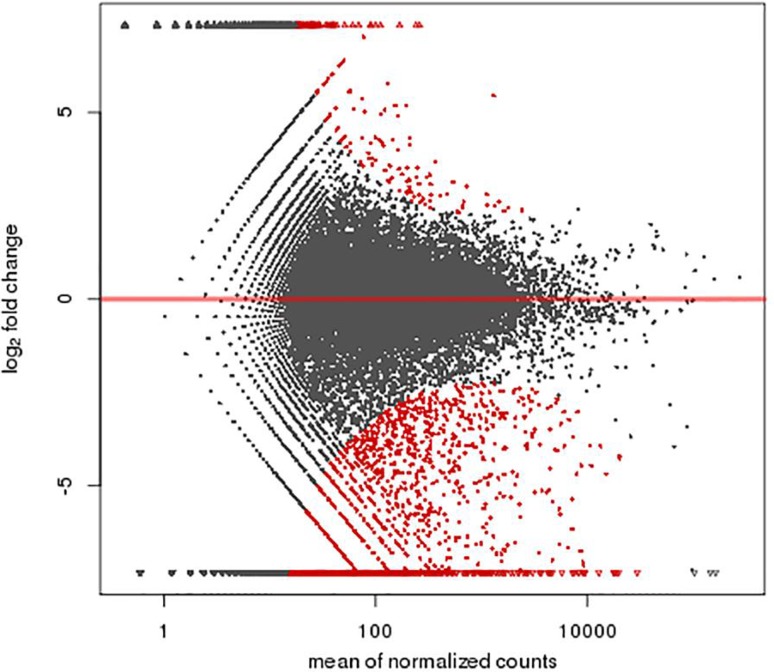
A total of 1,739 genes were differentially expressed between the co-blocking group and anti-B7 group, among which 166 genes were up-regulated and 1,573 downr-egulated. Abscissa: mean of normalized counts; Ordinate: log_2_ fold change.

### Differentially expressed genes

Table 3 lists the gene expression changes in the MR1 group and anti-B7 group. The expression of interleukin (IL)-10 was up-regulated, the expression of intercellular cell adhesion molecule-1 (ICAM1) did not change, and the expression of the others was downr-egulated. Between the co-blocking group and the other three groups, nuclear factor (NF)-kB, IL-1β, IL-1α and IL-2 were down-regulated, the expression of IL-10 was up-regulated, and the expression of interferon-γ (IFN-γ) did not change.

**Table 3:** Comparisons of gene expression among different groups. Down: gene expression was significantly down-regulated, compared with any two groups (p<0.05). Up: gene expression was significantly up-regulated compared with any two groups (*p*<0.05) -: change in gene expression was not significant between any two groups.

Gene name	GenBank No.	Gene expression comparison among different groups				
		MR1 and control group	Anti-B7 and control group	Co-blocking and control group	Co-blocking and MR1 group	Co-blocking and anti-B7 group
Interferon gamma (IFNG) mRNA	NM001081991	Down (*p=*0.0067)	-	-	-	-
Receptor activator of NF-kB ligand	AY753407	Down (*p=*0.010821)	Down (*p=*0.01097)	Down (*p=*0.01024)	Down (*p=*0.01031)	Down (*p=*0.01051)
Intercellular adhesion molecule 1 (ICAM1) mRNA	XM008250743	-	-	Down (*P=*0.01095)	-	-
Interleukin 1 beta (IL-1β) mRNA	NM001082201	Down (*p=*0.00017)	Down (*p=*0.00015)	Down (*p=*0.00011)	Down (*p=*0.00014)	Down (*p=*0.00014)
Interleukin 1 alpha (IL-1α) mRNA	NM001101684	Down (*p=*0.00029)	Down (*p=*0.00022)	Down (*p=*0.00018)	Down (*p=*0.00017)	Down (*p=*0.00020)
Interleukin 2 (IL-2) mRNA	NM001082064	Down (*p=*0.00107)	Down (*p=*0.00137)	Down (*p=*0.00095)	Down (*p=*0.00100)	Down (*p=*0.00101)
Interleukin 10 (IL-10) mRNA	NM001082781	Up (*p=*0.00501)	Up (*p=*0.00487)	Up (*p=*0.00421)	Up (*p=*0.00453)	Up (*p=*0.00491)

This experiment required a fold-change value of ≥2 for a gene to be considered differentially expressed. As shown in Table 4, IFN-γ was differentially expressed only in the MR1 group, NF-κB was not differentially expressed, and ICAM-1 was differentially expressed only in the co-blocking group. Furthermore, IL-1, IL-2, and IL-10 were differentially expressed. Compared with the other monotherapy groups, the IL-1, IL-2, and IL-10 genes exhibited significantly different expression.

**Table 4:** Summary of differentially expressed genes among the four groups.

Gene name	Fold-change (MR1vs control)	Fold-change (anti-B7 vs control)	Fold-change (co-blocking vs control)	Fold-change (co-blocking vs MR1)	Fold-change (co-blocking vs anti-B7)
Interferon gamma (IFNG) mRNA	6.1642055	-	-	-	-
Receptor activator of NF-kB ligand	0.1601425	0.0172554	1.3507824	1.0356758	1.1198796
Intercellular adhesion molecule 1 (ICAM1) mRNA	-	-	4.629242	3. 309105	3. 0141625
Interleukin 1 beta (IL-1β) mRNA	3.392174	3.567923	9.978432	6.0154725	6.0275084
Interleukin 1 alpha (IL-1α) mRNA	4.906946	4.136102	7.597166	5. 0150641	5.0234321
Interleukin 2 (IL-2) mRNA	2.1243224	2.6532710	3.2407665	2.1103629	2.7974085
Interleukin 10 (IL-10) mRNA	3.1750364	3.0018464	4.1170452	2.2259462	2.5397026

A differentially expressed gene was only identified when its fold-change value was ≥ 2.

## Discussion

An increasing number of studies have revealed costimulatory signaling pathways in corneal allograft rejection, such as CD28-B7, CD40-CD40L, and B7-ICOS (Ye *et al.*, 2014). Among these, the CD28 and CD40 signaling pathways are important in the field of clinical allograft, since they complement each other. Previous studies of organ allografts revealed that co-blocking of CD28-B7 and CD40-CD40L had a synergistic effect. Kirk *et al.* (1997) inhibited these two pathways in a kidney allograft model in rhesus monkeys, and found that the co-blocking effects were better than those obtained when blocking one pathway alone, and the survival period was extended to more than 150 days. In addition, these two pathways were independent. Simply blocking the CD40 or CD28 pathway reduced corneal allograft rejection.

CD40 and CD28 are transmembrane glycoproteins, while CD40L and B7 are their primary ligands, respectively. The binding of the ligand and receptor may initiate the costimulatory signal, thereby activating T cells to proliferate and differentiate into effector cells and produce effects (Kolly *et al.*, 2007). Howland *et al.* (2000) verified that the response of T cells to antigens in the absence of CD28 and CD40. CD28 plays an important role in initiating T cell responses, whereas CD40 maintains the reactions. The inhibition of CD28 prevents the differentiation of Th2 cells, and inhibiting CD40 may be more efficient for the production of Th1 cytokines. Therefore, blocking the CD28 pathway may inhibit T cell activation, while blocking the CD40 pathway may inhibit Th1 cell differentiation and maintain T cell reactions.

In the present study, the corresponding antibodies were injected under the conjunctiva of the rabbit high-risk corneal allograft model, and these competitively bound the ligand to inhibit the aggregation of the signaling complex and terminate the activation of T cells. Thus, the activation and maintenance of the immune response were inhibited. The ultimate goal was to avoid T cell-mediated immune rejection.

Our experiments confirmed that co-blocking CD28-B7 and CD40-CD40L costimulatory signals significantly inhibited high-risk corneal allograft rejection, and graft survival time was longer than in the single block mode. Furthermore, the present study compared corneal tissues in the control group, MR1 group, anti-B7 group, and co-blocking group at the gene level, as we performed RNA sequencing to screen for differentially expressed genes. Thus, the impact of co-blocking CD28-B7 or CD40-CD40L costimulatory signals on high-risk corneal rejection was examined. This experiment focused on immune rejection-related genes, such as IFN-γ, NF-κB, ICAM-1, IL-1α, IL-1β, IL-2, and IL-10.

IFN-γ is mainly produced by activated Th1 cells. It functions as an effective monocyte/macrophage inducer to induce the expression of major histocompatibility complex-II-class antigens on the cell surface, and strengthen antigen presentation and specific immune recognition processes. King *et al.* (2000) found that rats without cornea surgery or corneal graft rejection revealed no IFN-γ mRNA expression in the corneal graft, and that the IFN-γ mRNA level in the graft significantly increased by 9-13 days after surgery when allograft rejection occurred, suggesting that IFN-γ plays a role in corneal graft rejection. Skurkovich *et al.* (2002) used an anti-IFN-γ antibody in human penetrating keratoplasty. They found that visual improvement and graft opacity were better than before surgery, and no change was observed after three months of surgery, indicating that the anti-IFN-γ antibody significantly suppressed corneal graft rejection. In the present study, the IFN-γ gene revealed no significant change in expression, probably because when immunity occurred or the corneal graft was removed 28 days later, the IFN-γ mRNA level had already decreased, and could not be detected at the gene level.

NF-κB is a nuclear transcription factor with broad roles and present in nearly all cells in humans. Under normal conditions, NF-κB exists in a non-active form in the cytoplasm. When cells are stimulated by specific factors, such as IL-1 or tumor necrosis factor-α, the inhibitory protein is released and NF-κB enters the nucleus. NF-κB can regulate the expression of specific genes involved in inflammation and the immune response, such as cytokines (IL-2, IL-12, vascular endothelial growth factor, and tumor necrosis factor-α), adhesion molecules (ICAM-1 and VCAM-1), and chemokines (IL-8, MCP-1 and RANTES), which affect immune responses (Afonina *et al.*, 2015; Proto *et al.*, 2015). The proliferation and differentiation of T cells, B cells, lymphocytes, and dendritic cells also requires the participation of NF-κB, and it plays an important role in humoral and cellular immunity.

Cell adhesion is a condition of inflammation, immunity, and neovascularization, and ICAM-1 is an important cell adhesion molecule. ICAM-1 can induce leukocytes to adhere to activated vascular endothelial cells, thereby promoting leukocytes to migrate towards the graft and mediate interactions between T cells and the corresponding target cells. In addition, ICAM-1 can stimulate antigen-presenting cells, thereby activating T lymphocytes to differentiate into effector T cells. ICAM-1 can also help transfer the activation signals of Langerhans cells to T cells. After gathering different leukocyte subsets towards the inflammation sites, ICAM-1 can initiate or enhance inflammatory and immune reactions (Demmers *et al.*, 2015). Thus, ICAM-1 is closely correlated to corneal graft rejection and can effectively inhibit the synthesis and secretion of ICAM-1, which has become an important objective in anti-rejection treatment.

IL-1 is a cytokine that functions in the early stage of infections and is extremely important for promoting corneal allograft rejection. IL-1 is expressed in most nucleated cells, as well as in the normal cornea. There are two types of IL-1, IL-1α and IL-1β that have similar functions and can bind to the same receptors. IL-1β is the dominant form. BenEzra *et al.* (1990) reported that IL-1 strongly influences the formation of new blood vessels, and copy number variation affects the occurrence of corneal rejection. Under antigen stimulation, IL-1 levels are significantly increased to regulate the acute inflammatory phase, produce chemotaxis, and activate inflammatory cells and antigen-presenting cells, thereby activating T cells and B cells. In addition, collagenase levels and adhesion molecule expression are increased.

According to the gene expression results, IL-1α, IL-1β, ICAM-1, and IL-2 are down-regulated in the co-blocking group compared to the monotherapy group. These genes promote corneal allograft rejection. Thus, along with the weakened expression of these genes, rejection was effectively suppressed. IL-10 expression was found up-regulated in the co-blocking group when compared to the monotherapy groups, causing inhibitory effects on corneal allograft rejection. Thus, along with the enhanced expression of IL-10, rejection was significantly reduced, and graft survival time in the co-blocking group was significantly longer than that in the other three groups. NF-κB and IFN-γ did not exhibit any significant changes in expression level, which should be further evaluated by selecting different time points after removing the corneal allograft.

The following limitations of the present study must be acknowledged. First, due to the small sample size and low number of selected genes, further comparisons and analyses need to be conducted in studies with a larger sample size and additional genes. Second, because of the differences between rabbit and human eyes, the test remains a controversial method to assess the ophthalmic drug action in humans. Finally, although the co-blocking of CD28-B7 and CD40-CD40L costimulatory signals can prolong corneal allograft survival time, the anti-rejection effects of co-blocking of CD28-B7 and CD40-CD40L over longer periods require further study. Despite these limitations, this is one of the first studies to reveal the impact and mechanism of co-blocking of CD28-B7 and CD40-CD40L costimulatory signals on corneal allograft rejection.

In conclusion, the results of the present study revealed that the co-blocking of CD28-B7 and CD40-CD40L costimulatory signals can significantly reduce the expression of genes that promote corneal allograft rejection. At the same time, the inhibition of corneal allograft rejection gene expression was significantly enhanced.
